# COVID-19 ethics: unique aspects and a review as of early 2024

**DOI:** 10.1007/s40592-024-00199-x

**Published:** 2024-07-13

**Authors:** Wayne X. Shandera

**Affiliations:** Baylor College of Medicine, Houston, TX USA

**Keywords:** COVID, Provider ethics, Autonomy, Distributive justice, Vaccine hesitancy, Research ethics

## Abstract

COVID-19 presents a variety of ethical challenges in a set of arenas, arenas not always considered in past pandemics. These challenges include issues related to autonomy, distributive ethics, and the establishment of policies of equity and justice. Methods are a literature review based on regular editing of an online textbook during the COVID-19 outbreak and a literature review using key ethical terms. Patients are confronted with new issues related to autonomy. Providers need to expand their concepts of ethical issues to include decisions based on proportionality and public health ethics. The public health sector needs to assess the beneficence of alternative modes of disease control. The research community needs to redefine the concept of informed consent in emergent conditions. All elements of the medical spectrum—physicians, scientists, and the community-at-large including the pharmaceutical industry—need to consider the multifaceted methods for preventing future pandemics. This will require giving particular emphasis to public health funding and ending the documented discrimination that exists in the provision of proven therapies. The developing world is especially at risk for most of the ethical issues, especially those related to equity and justice. The ethical issues associated with the COVID-19 outbreak are not unique but provide a diverse set of issues that apply to patients, providers, social groups, and investigators. The further study of such issues can help with preventing future outbreaks.

## Introduction

The advent of the COVID-19 outbreak presented the world with a new pathogen, the SARS-CoV-2 virus, and an illness associated with an unusually high morbidity and mortality. By early December, 2023, over 772 million persons worldwide had acquired the infections and nearly seven million had died. (WHO 2022) The initial reports of high infectivity before symptoms were manifest suggested that the pathogen has some unique characteristics, also evident in the unusual number of long-term complications suffered by a small but significant percentage of COVID-19-infected patients.

Because each pathogen shows unique clinical aspects, the ethical aspects associated with SARS-CoV-2 infection may also be unique. This review describes the associated ethical aspects of COVID-19 infection. The multifaceted nature of ethical issues associated with COVID-19, or more broadly, SARS-CoV-2 infection, will be addressed from the perspective of the history of pandemics, the individual and the prospective patient and attendant family members, the provider including nursing and paramedical personnel, the public health system, the investigative teams, and finally society-at-large with particular attention to prevention of similar future pandemics and the problems of the developing world. Most ethical issues belong in multiple categories and Table 1 lists some examples of ethical issues associated with COVID-19.^1^

**Table 1:** Examples of major ethical principles challenged by the COVID-19 outbreak

	Principles	Examples
A	**Equity**	**Everyone has the same opportunity to acquire good heath** Blacks, Native Americans, diabetics are at high risk for COVID compromising their acute and future health
B	**Justice**	**The same regimens are uniformly available** In the developing world access to vaccines and pharmaceutical are severely impaired
C	**Autonomy**	**Patients can accept to reject a course of medical action** In acute situations decisions are made arbitrarily by ER doctors
D	**Transparency**	**Medical decisions are made with honesty, reliability and integrity** Some medical trial regarding hydroxychloroquine were not carried out ethically
E	**Solidarity**	**Benefits and obligations are carried out equally throughout society** Some vaccine trials benefited primarily those in the developed world
F	**Good science**	**The principles of scientific investigation, such as Koch principles are carriedout and used to make decisions** Leaders touted unproven forms of therapy
G	**Concern for the disadvantaged**	**The underprivileged, the impoverished are given particular concern**
H	**Duty to treat**	**Providers may not discriminate against patients based on disease or medical status** Shortages of supplies and nurses were common in many areas during the outbreak
I	**Proportionality**	**The benefits of a policy outweigh the burdens** Do lockdown measures prevent disease more than deflate the economy?
J	**Beneficence**	**The best interests of patients are given primary concern** Triage decisions may make beneficence difficult
K	**Nommalfeasance**	**Doing no harm is a mainstay of medical care** Appropriate therapies are not universally espoused by providers

## COVID-19: is it unique relative to the history of other pandemics?

Whether an outbreak can be defined as a pandemic is often arbitrary with the differences between the endemic, epidemic, and pandemic status of a pathogen dependent on knowledge of disease dynamics, the background rates of a disease, and the validity of global reporting. Endemic diseases occur when an established background rate can be defined, epidemics are defined by a statistical surge over such background endemic rates, and pandemic diseases are those which occur over a large geographic area within a defined time course. It is often stated that one case of severe disease where zero are expected (for example, botulism) is an epidemic. Defining a new entity such as a pandemic requires the luxury of time before its nature is fully understood, and this time course depends on the incubation period, severity, and reporting circumstances.

The global or pandemic nature of a given pathogen is certainly not unique. Global pandemics include most prominently the influenza outbreak of 1918. during which time the identification of the actual pathogen did not occur until 1934 when the influenza virus was isolated (Barry 2004), smallpox in many historic episodes; and tuberculosis whose widespread endemic nature does not fit the actual definition of an acute pandemic. The outbreak of COVID-19 is certainly the first publicly described pandemic to surface during the Internet Age, with the development of the World Wide Web in 1989 at CERN through the work of Tim Berners-Lee (McPherson 2010) and its wide dissemination only in the early 1990s. Other more recent clinical infectious diseases, such as Zika, Ebola, and chikungunya virus infections were localized in their endemicity (Brazil, West Africa, and SE Asia, respectively). The 1999 influenza pandemic, which was first observed in Mexico City, comes closest to being a pathogen of the Internet Age, but the already prevalent influenza vaccines allowed for rapid control of this outbreak.

The attribute of SARS-2 showing high infectivity before clinical disease is not unique in clinical infectious diseases. The diverse characteristics, including epidemiologic ones, of pathogens in relation to clinical ethics were emphasized in a review several years before COVID-19 was identified. (M. J. Smith and Silva 2015)^2^ The unique aspects of the epidemiology of COVID-19 are outlined in Table 2.

**Table 2:** Unique aspects of the COVID-19 pandemic

(1) A high rate of infectivity before manifestation of clinical disease (Alene et al. 2021)
(2) An unusual high pulmonary morbidity (Johnson et al. 2020)
(3) A broad range of age-associated disease^a^
(4) A development in some cases of multisystemic manifestations including a an inflammatory state in children (MIS-C) (Santos et al. 2022)
(5) A tendency in some infected persons to evolve into a chronic disease (often referred to, first by the British as “long COVID”) (Akbarialiabad et al. 2021)
(6) A high influence of social media attendant with both positive and negative aspects; (Venega-Vera et al. 2020)
(7) Past issues related to travel and border restrictions^b^ (Le et al. 2022) (Moodley et al. 2022)
(8) A history of employment restrictions associated with infected personnel including the undefined impact of stay-at-home or work-from-home policies. (Howe et al. 2021)

^a^Shared with the somewhat more bimodal respiratory syncytial virus) (“*COVID-19 Stats:* COVID-19 Incidence,* by Age Group† — United States, March 1–November 14, 2020§” 2021)

^b^Ironically bans were set up after South Africa released Omicron variant genome data. (Le et al. 2022)

Other pathogens that can be infectious prior to onset of symptoms include measles (infectious for a week before the typical rash), varicella (infectious for 2 days prior to manifestations), and rubella (which can be infectious for a week before symptoms are present). The malady known as fifth disease (erythema infectiosum) can be infectious for a week before symptoms occur, and mumps can be infectious for four days before the classical swelling of parotitis is manifested. All of these are diseases largely of children, but two adult diseases that can also be infectious before symptoms occur are bacterial meningococcal meningitis (for a week before symptoms) and hepatitis A (for two weeks before jaundice). (Papadakis et al. 2020), (Hay 2018) Thus, this aspect of the COVID virus, which is infectious 1–2 days before symptoms, is not unique but its presence in a globally-dispersed agent makes the clinical ethical issues especially salient.

Many of the historic issues of pandemics are outlined in several books that address pandemics.^3^,^4^ The issue of distributive justice—that is, who should receive the vaccine in times of scarcity—is seen repeatedly in the outbreak. The author Anne Lykkesov (Ethical Theory and Moral Practice) from Copenhagen criticizes Bramble’s theses as reductionist and considers many of his arguments as without proof. (Lykkeskov 2021) Bramble’s urgency however on another aspect—the effect of the pandemic shutdowns on the education of youth—is supported by data from the US (that emerged in October, 2022) that the National Center for Education Statistics (US based) show a plummeting of reading and mathematical skills among US grade school students ((Hoofman and Secord 2021)), with especially significant plummeting in mathematical skills comparing 2022 and 2020.

These concerns lead to the questions whether the COVID outbreak is associated with a new spectrum of medical ethical issues. The author Jennifer Blumenthal-Barby argues that the ethical issues developing during COVID-19 era are not so far unique enough to warrant developing a new field of medical ethics. (Blumenthal-Barby 2020). Her conclusions are described with the hope of providing the best preventive scenarios for the future. In a review describing the historic pandemic precedents to COVID-19 including smallpox, influenza (in its many variants that became specified as more was learned about the pathogen), HIV/AIDS and Ebola, Sampath also points out the multifaceted impacts of pandemics including economic, social, and also mental health issues. (Sampath et al. 2021).

Public perception of the COVID-19 outbreak also identifies from surveys the low priority for resource allocation among forensic psychiatric and correctional populations. (Géa et al. 2022) The extent of stigmatization for psychiatric populations is recognized by a Canadian group (Chalmers et al. 2021) to include the genetic basis of other potentially discriminated against diseases such as schizophrenia, and the hazards such patients may face in the allocation of scarce resources.

Do the unique aspects of the COVID-19 pandemic extend to the developing world? Early data in the COVID-19 outbreak suggested that the developing world/LMIC (lower and middle income countries) were at a lower risk for infection, attributable to the window of preparation with early cases largely from the developed world and the lower mean age among residents of the LMIC. Early suggestive data that cold weather promoted survival also suggested the developed world is at lower risk for COVID and also perhaps genetic factors, including the higher prevalence of antibodies to galactose alpha-1, an enzyme which may downregulate the immune responses of COVDI-19, (Hodžić et al. 2020) These are reviewed in a rheumatologic report. (Hodkinson et al. 2020).

Despite such theories that suggest the developing world is not necessarily at great risk of pandemic instability, a perception among many societal funding agencies that prompted a response to containing COVID-19 in the developing world. This was reflected from the start when over the first year of the outbreak the World Bank Group committed over $200 billion to fight the pandemic. (World Bank 2023) The countering issues inducing such as responses include the excess morbidity from infectious diseases in the developing world, the increasing prevalence of chronic diseases, and the impaired social and economic structures in many developing countries. These issues suggest to some authors that is imperative that the developed world heed the potentially devastating impact of COVID-19 in LMIC. (Bandyopadhyay and Vadlamannati 2022) The impact of COVID-19 is documented in Latin America, for example, in one article which described the effect on disease status of “abysmal differences” between these economies and those of advanced economies. (Proaño 2020).

In summary the COVID-19 pandemic has many distinct aspects, outlined in Table 2. Despite these aspects, it is not fully evident to many authors that the ethical issues associated with the outbreak are unique. The challenges in the developing world include recognition that there exists both a decreased and an increased risk for COVID-19 infection in developing countries.

The remainder of the paper will address specific ethical issues from different perspectives.

## The prospective patient and clinical ethics

The first prominent clinical ethical issues at the start of the pandemic was the need of patients to accept rationing of respirator use among those infected with severe pulmonary disease. This often involved the use of arbitrary scales for ventilator use, such as one devised in Minnesota, a scale criticized for exacerbating known inequities. (Kesler et al. 2021). Other clinical ethical issues included the rapid recognition that particular races were more likely to be affected. This was evident in particular when New York City was impacted and the selective infection of Black and Hispanic residents was published. (Marcello et al. 2020). Whether immunogenetic factors are involved is still under study. (David et al. 2022).

Autonomy is a cardinal issue with many manifestations. It was the basis for recommendation of a Texas governmental official advising that the elderly show a willingness to die to support the economy for the next generation, (Knodel 2020), Other manifestations of autonomy include the reluctance of patients to take the proven therapeutic agent nirmatrelvir and ritonavir (Paxlovid^®^) and to instead support unproven therapies (hydroxychloroquine, ivermectin). While the efficacy of nirmatrelvir/ritonavir was established early among the elderly and those with risk factors for hospitalization and death, the over-all efficacy is not fully established when the treated population includes fewer less at-risk populations and individuals from a variety of geographic background (Chew et al. 2023) Nonetheless, the medical establishment views as outlined by the Infectious Disease Society of America (Bhimraj et al. 2022) supported agents such as Paxlovid^®^ and flatly repudiated many alternative types of popular medications. The libertarian and autonomous views of many patients also were the basis for the repudiation of legal mandates (Parker et al. 2021).

Autonomy is one of the least represented ethical issues in reviews of COVID ethics, the most commonly cited being justice and nonmaleficence. (Seyferth et al. 2022) A particular area where autonomy is potentially compromised is among elderly individuals with the use of digital sources, many of whom are not computer savvy and potentially must compromise confidentiality in seeking assistance with computer-related issues. (van der Graaf et al. 2020) Such digital exclusion of the elderly often exacerbates social isolation and loneliness. (Seifert et al. 2021).

Another ethical issue described by the poignant and perhaps unique aspect associated with COVID was the isolation of the elderly in nursing homes. Families were often prevented access to dying loved, ones and in turn the elderly were deprived of close contact with friends and family members. Similar issues arose in the United Kingdom in care home facilities. (Cousins et al. 2021) It is hard to think of another contemporary infectious disease where such stringent policies affected patients and families so intimately. (Abbasi 2020) Patients with dementia were particularly impacted by the social isolation which COVID-19 imposed upon them, and in a review of 15 papers, one team from the UK and India reported that in 14 (93%) reports the onset of new behavioral or psychological symptoms existed (Suárez-González et al. 2021) Perhaps in Biblical times lepers (who are mostly now thought to have had psoriasis) were subject to such extreme isolation. Similarly smallpox and tuberculosis (in its many historic names, phthisis, consumption) often resulted in quarantine or prolonged isolation.

Employees of long-term care have a set of ethical challenges related to the moral distress of “carrying a load,” as well as the risks of burning out which may be amplified by their unique circumstances, and the feeling that one should “work through problems” as part of a “duty to care.” (Boamah et al. 2023).

Televisits are felt by some to be a significant way COVID is changing the practice of medicine. (Tepper et al. 2020) The ethical issues associated with telehealth have received little attention, and in one review the main concerned of authors were autonomy, professional-patient relationships, nonmaleficence, beneficence and justice. Further studies and guidelines are needed in the area of telehealth, evident now since the COVID-19 pandemic brought telehealth to the forefront. (Keenan et al. 2021) Those who use telehealth show especial problems with distributive ethics (Bejarano 2022)^5^ The elderly are less likely to feel comfortable with such visits; those who do not have phones or computers and thus limited access to care present additional ethical issue.

The disproportionate needs for care of COVID patients set up a complex mixture of ethical issues prompted by characteristics of the outbreak. Delayed operations reduced availability of facilities for chronic diseases from cancer to chronic disease such as multiple sclerosis, to the impaired tropical diagnoses of malaria have all been documented in the COVID pandemic. Resources, personnel and materiel are modified in the era of COVID. (Muhrer 2021) Such issues overlap with social and preventive ethical issues and beg the issues of clinical triage. Triage itself is an issue that is insufficiently studied. Early in the outbreak the need for better triage criteria were described, and gold standards for triage were complicated by inconsistent application of triage guidelines by the attendant officers. (Iacorossi et al. 2020).

Triage is also analyzed in its fulfillment of the criteria of distributive justice. In one review, the component criteria of utilitarianism is often at odds with that of egalitarianism, and the ancillary criteria of libertarianism and communitarianism are not always fulfilled by major triage systems. (Meier 2022) Other authors contend that the chief tension in COVID-19 ethics in the community is between utilitarianism and social justice, the former restrictive and the latter more expansive in its approach to social problems. (Bispo Júnior and Morais 2020).

Patients engaging in vaccine hesitancy also express autonomy. and this is the subject of many reviews, including analyses of special populations and individual countries Studies analyzed early in the outbreak documented high levels of hesitancy among Black Americans—despite their higher risks of acquiring COVID-19 and dying from COVID-19) (Bunch 2021)—and pregnant or breastfeeding women. General increases in acceptance occurred during the first year vaccines were available. (Yasmin et al. 2021) Early studies of global vaccine acceptance showed high variability, from 97% in Ecuador to 23.6% in Kuwait, and even variability among health care workers (HCW), for example, 78.1% in Israeli HCW but 27.7% among HCWs in the Democracy Republic of the Congo. (Sallam 2021).

Distributive justice issues are prevalent throughout the pandemic. The issues of distributive justice are nowhere more strikingly illustrated and poignant than when considering the predilection of patients from advanced economies to use healthy individuals in the developing world as a source for renal transplants., (Kute et al. 2021) Such distributive justice issues also are illustrated by developing world food insecurity, a consequence of increased violence, food fraud, alterations, and crime, complicated by labor shortages. (Panghal et al. 2022) Both procedural and distributive justice were reviewed in the need to face the inequities of Indigenous Peruvian tribes during the COVID-19 outbreak, using the needs and aspirations of Ashaninka and Shawi peoples. (Chicmana-Zapata et al. 2023).

In the developing world, phone surveys report that females, the young, the less educated, and urban residents are all overrepresented among those most economically impacted by the pandemic. (Kugler et al. 2023) Also, the prevalence of new-onset diabetes from COVID-19 is statistically more prevalent in the developing world than in the developed world (19.5% vs 9.4%). (Junaid et al. 2023) Such findings expose the differences in equity for the developing world.

In summary, a whole spectrum of clinical ethical issues apply to the individual from autonomy (what decisions should patients be allowed to make in the absence of proof, for example), to distributive ethics (the clinical course of a poor immigrant as one who is at high risk of COVID associated clinical disease and complications). The providers of COVID care are intimately involved in these decisions and this is the next subject for review.

## Provider ethics

This section will consider the ethical issues that providers face including equity and proportionality issues, and the concept of duty-to-care, the unique challenges faced by certain professional groups including nurses, neurologists, pulmonologists, and surgeons. A few comments about providers in the developing world complete the section.

The list of ethical issues confronting the provider of quality care in the COVID-19 pandemic is long. In one extensive analysis, the most commonly listed issues were equity, reciprocity, transparency, justice, duty to care, liberty utility, stewardship, trust, and proportionality. (O’Sullivan et al. 2022).

Perhaps the most prominent ethical issue relates to the lack of equity and justice. Structural racism and injustice is a product of racial inequalities, and the conventional nature of racism needs recognition as “un-extraordinary” to expose its evil essence. (Ganguli-Mitra et al. 2022) The complexity of the racial predilection and the inherent racism in society exposed by the outbreak is present at many levels. One never considered is the impact in academia. Minority faculty, especially Black faculty are often required to carry out a disproportionate amount of diversity, equality, and inclusion work in their academic careers, a trend referred to as a “minority tax.” The interface of these trends mean that minority faculty are not only at increased risk for COVID-19 and but also face work limitations in their need to support they give for diversity, equity and inclusion programs. The sad consequence is a loss of minority representation in academic programs. (Layne et al. 2023).

The importance of protecting all health care providers, especially with core ethical issues, is evident in the plea and poignant need for this in a reflection from an Italian physician at the height of the outbreak in his country who stated that “only with transparency and inclusivity can public trust and cooperation be achieved.” (Rosenbaum 2020) The limited supply of personnel protection equipment (PPE) also exposed the risks of providers and the difficult issues they faced early in the outbreak.

The concept of duty-to-care can become quite encompassing. Besides the traditional aspects of duty to care during an outbreak, elucidated vividly during the HIV-1 outbreak, providers are confronted with a host of needs. Providers and administrators are obligated to maintain standard-of-care facilities, including personal protective equipment, as well as protocols and access to remote connection technology. An obligation exists to improve equipment and facilities as they become available. (Arabi et al. 2021) Providers during the COVID pandemic have had to avail themselves of several developments that, while not unique to this pandemic, did proliferate and become more readily used, including competency-based education, interprofessional education, and large-scale application of information technology to education. (Frenk et al. 2022).

This concept of duty-to-care evolved long prior to COVID, and articles regarding this concept appear in relation to influenza (Chalmers et al. 2021) and HIV (Halevy and Brody 1994) infections. This policy is not unique to American culture. (Freedman 1990) The concept of duty-to-care is tempered by the frequency with which providers are member of high risk groups (in particular age and diabetes but also underlying immunosuppressive states). The limited work force in many facilities is exacerbated when providers with at-risk conditions are excluded from the workforce. A sense of proportionality (is the benefit greater than an imposed burden?) is essential in making guidelines to determine who should be required to work. Physicians may also face the societal request for mandatory vaccination, a concept that has led in some areas to legal actions. (Parker et al. 2021).

Fins and Miller (2020) also emphasize the importance of proportionality in making decisions that protect health care workers. They emphasize using prudence in deciding what benefits patients most, as well as identifying what actions put one’s staff at risk. Other authors note the importance of using resources judiciously. (Hermerén 2012) The need to delineate duty-to-care in guidelines is also cited as important, in an article with a Canadian perspective. (Ruderman et al. 2006).

Some authors such as Malm feel otherwise and argue that the traditional duty-to-treat principles—including consent, implied consent, special training, reciprocity or social contract, and professional codes—do not adequately support the duty to treat (2008). In the end the authors place the responsibility upon society to find creative ways to employ an adequate number of health care workers in a pandemic.

Other authors also emphasize the need for physicians to navigate through moral dilemmas because, without clear guidance, lasting untoward career implications may ensue. Navigating through moral dilemmas, providers also need to keep abreast of medical and scientific development and learn the new terms associated with COVID, (Sampath et al. 2021) Terms such as “brain fog” and “long COVID” are new, and associated uniquely with the outbreak, and both are examples of colloquial terms that are not well-defined. “Brain fog” is one of three most commonly described symptoms with COVID-19, despite it being a poorly understood concept (Krishnan et al. 2022). The WHO has a broader definition of what it encompasses. “Long COVID,” a term first established by the British, is also in search of a strict definition and is also by and large a colloquialism but used often by clinicians and therapists in an attempt to define a more exact entity. (Sharma et al. 2022).

The provider perhaps most impacted early in the outbreak was the nurse, because of the amount of time spent with patients, the shortage of trained personnel, the supply chain deficiencies, the need often to be the only person present for dying patients. The variety of euphemistic terms, superheroes, essential workers, martyrs to a cause, does not begin to address the severity of the work performed by nurses at all levels, from bedside licensed vocational nurses, through nursing administrators attempting to keep a clinic or hospital staffed.

A nursing review from Thailand (Turale et al. 2020) emphasized and expanded early in the outbreak on these concepts and the resilience and stamina the nursing industry had to maintain while facing end-of-life ethics and diverse cultural ethics associated with most facilities in the world today. Nurses are particularly susceptible to one ethical issue: that of moral injury including regrets for ethical situations, feelings of guilt without fault, and existential suffering when administering pain, and the feeling of often not doing enough. While not unique to nurses, the frequent position of nurses in their utmost primary care role makes them susceptible to such “moral injury,” and it is felt that more research in this area is needed. (Čartolovni et al. 2021).

Neurologists have emphasized the short-term view that most physicians engaged early in the outbreak, with little attention to the long-term. This was due to the need to increase short-term survival for infected patients. Since the aged were at highest risk early in the outbreak, the authors emphasize, as do many others, the importance of prioritization, a concept dependent on the arena one is working with or describing. (Kim and Grady 2020) For example, a busy emergency room will triage based on objective parameters (oxygen saturation, viral signs, risk factors of patients), while an ICU might use a published scale to determine which patients should have priority regarding placement on a ventilator.(Ervin et al. 2020; Nasa et al. 2021).

Pulmonologists have been confronted from the onset of the outbreak with issues related to supply distribution, for example, of ventilators. A set of guidelines as mentioned above is used often to objectify the process of making such life-determining decisions, with any federal and state guidelines providing assistance in the US. (Dos Santos et al. 2020) Ventilator distribution as part of an ICU guideline SOFAM (Sequential Organ Failure Assessment) used nationally has been criticized as not addressing the differentially higher creatinine levels (which may not signify impaired renal function) among the Black community. (Schmidt et al. 2022).

Surgeons have suffered from a unique set of ethical issues. They are forced to make decisions and triage patients in a public health manner, with one author stating that surgeons now consider public health ethics over individual patient ethics. The need to delay many operations early in the outbreak, the need to use an excess of paternalism in dealing with dying patients, the need to withdraw some care in the early stages of the outbreak for fear of infecting staff or patients, are ethical issues that often do not come easily to the activist, doing-oriented surgeon. (Angelos 2020) Neurosurgeons are also self-critical of the need to establish treatment priorities, the risks of moral injury and associated medicolegal and financial uncertainties associated with their profession. (Tsermoulas et al. 2020).

An abundance of studies exist on provider issues from the developing world The nursing study from Thailand is outlined above.(Turale et al. 2020) Studies on the management of mild to moderate cases from the developing world include US sponsored studies in Pakistan using phone treatment with ancillary laboratory studies with successful results. (Alishan et al. 2022) The increased risk of HIV infection among blood products in developing world locales is the bases for urging caution for providers who use of convalescent plasma therapy. (Ferreira and Mostajo-Radji 2020).

In summary, the challenges that confront professional providers are extensive ranging from allotment decisions to reassessing the concepts of “duty-to-treat” and establishing the need for many providers to change their concepts from short-term care to long-term ethics more akin to those seen in the next section on public health.

## The public health perspective

The public health sector often addresses issues that individual patients or providers do not consider. Issues to be discussed include the effect of social media, questions of proportionality including access to vaccinations and antivirals medications, vaccination policies and hesitancy, and deciding on proper policies for mask usage, and decisions on closure of facilities and schools. These decisions entail balancing medical, economic, and legal issues.

The COVID-19 outbreak has amplified the effect of such social and economic factors and these are made all the more evident by the diverse effect of social media. (Venega-Vera et al. 2020). Social media has also exposed the need for society to establish a balance between restrictive isolation measures (schools, hospitals) and the ongoing activity of such facilities. It is also possible that social media has a disproportionate impact on the vulnerable and less educated members of society raising the issues of equity and discrimination, Social media incidentally is not all harmful and shows benefits in that it can be an ancillary tool in the classroom when physical distancing prevents close contact. Studies from Pakistan for example show such benefits in school including those with religious scholars. (Jogezai et al. 2021).

How to measure the effect of public health effects is a controversial and difficult issue.^6^ A review from early in the outbreak cautioned against considering only economic outcomes in assessing efficacy of therapeutics. Many of the disadvantaged populations already suffer from shorter mortality and excess health care costs and any assessment of efficacy must consider these cofactors when placing justice as a primary goal in distributing vaccines and medications. (Schmidt 2020).

The concept of proportionality is especially important in assessing pandemic outcomes. An example is determining public health policy by comparing adjacent geographic areas with respect to public policy. In Los Angeles, for the early years of the vaccination, masking was far more prevalent than in adjacent Orange County. Many residents of the latter felt that masks impose a burden greater than a benefit, pointing to the ethical principle of proportionality. It may be naïve to make comparisons between two counties when so many other variables differ including vaccine acceptance, demographic differences, as well as the prevalence of underlying disease states. Mask mandates in the US existed in 38 states at the peak of the pandemic and CDC guidelines stress that masks were important in the early stages of the outbreak in diminishing rates of COVID-19 acquisition. (Guy et al. 2021).

Lockdowns entail a whole set of ethical issues. The ethical implications associated with them are the basis of a review. (Zadey et al. 2021) The authors analyze a spectrum of qualities and emphasize the efficiency and utility of lockdowns. But lockdowns are attendant with personal restrictions and their importance among the public is often controversial. The authors concluded that nonetheless lockdowns are ethically justified.

A challenging concept elucidated by Franco in a BMJ editorial is the different attitudes society takes toward lockdowns versus vaccines. Lockdowns were accepted early in the outbreak. Vaccines at a later time showed much greater efficacy but were rejected by significant sectors of society. Should we not, the editorial states, prescribe mandatory vaccines for effective disease control? (Franco 2021) Frith advocates the use of big data studies to ascertain the efficacy of lockdowns and argues that the results of such findings should be incorporated using the principles of solidarity, reciprocity, and stewardship. (Frith 2020).

An interesting perspective on vaccination acceptance or rejection comes from a study of the Hispanic community in San Diego in which certain cultural attributes such as **familismo** (attachment to family values) and a newly instructed term, **aguantarismo** (an ability to endure the vicissitudes of life without complaining) can work toward both vaccine acceptance and vaccine rejection. (Sobo et al. 2022) Achieving equity in vaccination is thus often culturally determined, and despite the high barriers in many communities for vaccine acceptance, modes can be developed that can result in greater vaccine acceptance. Such measures include language, culturally sensitive material, availability, and transport. (Grumbach et al. 2021) The variable access and acceptance of vaccines is a matter of both equity and justice. (Privor-Dumm et al. 2023) The use of medical students and community organizations such as churches can help address misinformation and accessibility as shown in a project from Reno, Nevada. (Adler et al. 2023).

The principle of equity is a very frequently and extensively discussed topic in relation to the COVID-19 outbreak. The need to address equity was outlined early in the outbreak, in 2020 when an article in the NEJM recommended attention to four important “social determinants:” a need for universal food income, a system of unemployment insurance, and investment in community development through low housing credits. and socially conscious reinvestment programs. (Berkowitz et al. 2020).

The global impact of COVID-19 on equity is well illustrated in a 13-country review by Shadmi in which 15 areas are analyzed and the universal problems of poverty, isolation and impaired mobility are shown to be ubiquitous. (Shadmi et al. 2020) An editor of JAMA redefined “herd immunity” as the improvement of social determinants for all aspects of society to the point that there is a resistance to the spread of poor health. The only long-term solution is the abolition of racial and ethnic disparities in heath so that disease transmission is impaired as equity is achieved. The results is an eventual achievement of social medical justice. (Williams and Cooper 2020) Authors from China feel that the lack of attention to health equity resulted in perhaps a halving of disease control efforts during the early years of the pandemic. (Wang and Tang 2020).

Governments during pandemics may criminalize behavior that may expand or worsen control of an outbreak, (Lehman et al. 2014) although many of these laws persisted on the books through the present. (CDC 2022) This happened during the early years of the AIDS outbreak. ((Harsono et al. 2017) (Gostin 1989). A review by Sun looked at 51 English-language orders among 39 countries in seven world regions using the COVID-9 Law Lab database of 190 countries. Few orders complied with requirements of human rights obligations and human rights responsibilities. (Sun et al. 2022).

How does a public health specialist then study proportionality with respect to public health measures, especially when heterogeneous communities often contain diverse amounts of discrimination? The answers to this will come with more studies and more understanding of the effect of public health measures and recognizing that changes in proportionality will exist across both time and space. (Fins and Miller 2020) (Hermerén 2012).

Public health populations often forgotten are those with disabilities and migrant/immigrant populations. The COVID outbreak has disproportionately affected these populations. To adequately care for the disabled one must confront the norms for protecting such vulnerable individuals as well as infringements based on distributive justice that such persons often face. (Sabatello et al. 2020) The public health sector almost uniquely has an obligation to advance the health issues for immigrant and migrant populations (Machado and Goldenberg 2021).

The need of public health officials to communicate information early in the outbreak, when resource scarcity and uncertainty of scientific findings existed, was a dilemma. It is acknowledged that the concept of the precautionary principles, that is the need to do no harm, often limits the capacity of the public health official to effectively transmit information to the public. The confusion attendant with policies related to masking early in the COVID-19 outbreak is perhaps an example of this public health need to transmit information at a time of uncertainty. (Lowe et al. 2022).

A segment of the society that is exceptionally important is the pharmaceutical industry. In a comprehensive review, an American team outlines the principles of the industry in a global health emergency as optimizing vaccine production, engaging in fair distribution, providing a sustainable product, and establishing a system of accountability. The needs for tiered pricing, and the importance of distinguishing bilateral and multilateral contracts including those established by COVAX (the COVID-19 Vaccines Global Access) of the Vaccine Alliance, address the tension inherent in production, distribution, and equity in providing vaccines to the world. (Emanuel et al. 2021) The need for greater transparency in the pharmaceutical industry is outlined in a primer from the European Union but its principles are universal. (Webb et al. 2022).

Vaccine policy is an important area of public health policy. The presence of a considerable segment of society who refuses to be vaccinated delays an obliteration of the outbreak. The association of anti-vaccination attitudes with political persuasions and membership in certain politically biased cults has also complicated the ethical dilemmas associated with COVID. Should social media sources be allowed to restrict access to certain groups? Mandatory requirements for COVID vaccination may face court judgments which impair their implementation. (Franco 2021).

The complexities of personal and society attitudes toward mandatory vaccination and toward quarantines are based on personal views (a self or other oriented cultural bias) and national views toward long-term goals are reviewed in a complex study out of the University of Michigan. The best support for mandatory vaccination exists among individuals who believe in justice for self rather than justice for others and where national values such as uncertainty avoidance support such mandates. Those who advocate justice for others support such mandatory policies primarily when governments support long-term goals. The complex interactions of variables merit study in assessing vaccine hesitancy and other prevention policies. (Lucas et al. 2022).

A nursing view also addresses the issue of mandating vaccinations (Maneze et al. 2023) The authors recognize the balance between autonomy for health care workers and the right to safe and quality health care by the public. Vaccinations, the authors cite, are not a panacea for safety. Access to well-informed and reliable guidelines is important, as is support for vulnerable health care workers.

Combating vaccine hesitancy is difficult. A host of methods encourage vaccine acceptance. Appointment reminders and opt-out scheduling systems are highly effective. Infographic and multimodal presentation are moderately effective. Financial incentives, which include the unusual Vax-a-Million cash lottery in Ohio, appear to be effective but with variability based on the jurisdiction. A thorough review of financial and nonfinancial interventions is available. (Terrell et al. 2023) A multistage digital intervention program appears to be an effective way to address hesitancy using motivational intervening principles. (Knight et al. 2021).

Particular populations in need of directed programs include those aimed at the homeless who accept vaccines more when provide with a mean of housing stability. (Ahillan et al. 2023) Refuges, immigrants and migrants tend to all be under immunized, (Daniels et al. 2022) The immigrant population is at risk for COVID-19 in many encounters with health and government officials: during Immigration and Customs Enforcement (ICE) enforcement actions, at ICE detention sites, and during deportation. (H. V. Miller et al. 2020).

Other at risk groups for vaccine hesitancy include pregnant women, Pregnant women among high income countries, the undereducated, and multiparous women tend to be less accepting of vaccines, (Bhattacharya et al. 2022) Members of the LGBTQ community are underrepresented in studies. (Garg et al. 2021) Attendants at fertility clinics score high levels on a client mistrust index and this mistrust correlates with vaccine hesitancy. (Kassi et al. 2022).

The developing world is subject to these same challenges. These challenges are associated with infrastructure, financing, transparency and health care management. These issues have been described in an article from India and these descriptions all support the need for developing world health care structure to be improved. (Malik 2022) It is hoped that the increased use of mobile health and telemedicine will benefit the public health systems globally but especially among LMIC. (Filip et al. 2022).

The influence of social media is very evident in the developing world. In developing countries the impact on the poorer and needier aspects of society is disproportionate. Such ethical issues are related to discrimination and to limited resources available in much of the world. The differences include selective availability of medications and vaccinations (the latter has been referred to as “vaccine apartheid” in the Lancet). (Bajaj et al. 2022) \

The poor correlations between national resources and the amount spent on public health exists in the developing world and is explained in a narrative review by Hasan et al. The importance of public health expenses in improving health indices is emphasized, the authors comparing the recent outcome experiences of Bhutan favorably with those of India. (Hasan et al. 2022).

A concern that providers report from the developing world is the high frequency of slums and the predilection of the SARS-2 virus to spread more readily in high-density areas. Thus some of the most vulnerable populations are in the developing world, especially in the very large cities of Africa and Asia. (Sahasranaman and Jensen 2021).

Patients who reside in the developing world are at an increased risk for SARS-2 infections based on lung disease from exposures to air pollution Studies show that an increase of 1 μm^3^ in PM_2.5_ is associated with an increased percentage in COVID-19 death rates, a phenomenon confirmed in multiple nations from China through Indonesia. (Chen et al. 2021).

Another factor disproportionately affecting lower and middle income countries is corruption. In a review of factors that increase the potential for risks, a Canadian group of investigators found that shortages of health products was a major factor increasing the potential for such corruption. Products affected included vaccines, diagnostics, personal protective equipment and controversial medication such as chloroquine. (Griffore et al. 2023).

One mode of responding to the scarcities and need for better transparency is the presence of a multistakeholder initiative known as Access to COVID-19 Tools Accelerator (ACT-A) which can encourage greater pandemic preparedness by transparent and accountable governance. (Moon et al. 2022) A Latin American and Spanish group of thinkers believe society should incorporate the concepts used in Transitional Justice (including civic trust, reconciliation, democracy, transparency, solidarity and social inclusion) as a means of addressing the social inequalities among health systems. (Rodríguez Reveggino and Becerra-Bolaños 2022).

Some analysts feel that the wealthy are hoarding vaccines. The principle of distributing vaccines to the less developed world is the subject of an analysis from China which suggests replacing traditional Kantian ethics, where the wealthy often feel obliged to give, with a humanitarian system for global justice based on actual needs and modeling of data. (Li et al. 2021).

In summary the diverse set of ethical issues that public health faces range from distribution ethics for the underserved to the controversial issues of mandatory measures which often conflict with societal libertarian views.

## Research ethics

The issues associated with the origin of the virus and the likelihood that a pathogenic virus was released from a laboratory raise a whole spectrum of ethical issues. But with the unprovable nature of such issues beyond a certain level, this topic will not be discussed further.

Research on COVID-19 patients, while paramount, is associated with a new set of challenges, ranging from the capacity of requests among review committee to special considerations associated with the pandemic. These include the need for expedited services, and in many situations the use of deferred informed consent. A Chinese team produced a review of such guidelines based on their experience with the outbreak and urged the establishment of new systems that address the sheer number of ethical reviews and establish methods for periodic revision of guidelines. (Ma et al. 2020) Research on pregnant women is a particularly important issue and embraces the areas of inclusivity, unique aspects of monitoring, and assurances that appropriate testing is taking place. (Mourad et al. 2020).

The concept of “deferred ethics” in the setting of COVID is receiving significant attention itself. One review (van der Graaf et al. 2020)) points out how often, especially early in the outbreak, that intubation had to be performed in the absence of informed consent, and that heretofore informed consent in the setting of pandemics has not been well studied. Such ethical principles apply to clinical and research management. The authors emphasize that in the research sphere, requisite conditions are numerous. These include an emergent condition, an incapable patient, unavailable legal representatives, and a sense of proportionality with respect to risks and benefits, timeliness, absence of other study populations, no advance objections, and the approval of an ethics committee.

An area under investigation is COVID-tracking apps (CTA) which typically engage in proximity and contact tracing. They are associated with their own arena of ethical issues including variable effectiveness and technical problems. Security is an issue with the fear of diverted use of private material. Distributive justice is evident as such CTAs are found disproportionately among those with the resources to buy them. (Klar and Lanzerath 2020).

The use of apps is seemingly tolerated in societies that do not protect civil liberties as much as in the West, such as China from early in the outbreak on. Parker also outlines the ethical concerns attendant with the use of contact-tracking apps such as the above stated need for security and privacy and the maintenance of professional ethics. He also emphasizes the need for guidance regarding decisions on how to handle data at the end of the epidemic. The principles of equity, justice, consistency, and case compassion are all needed and if handled appropriately may assist with future epidemic settings. (Parker et al. 2021).

Research on vaccination policies often entails the development of national vaccines of highly variable efficacy and begs the establishment of equity and justice in the field of vaccine research and production. This requires the support of dedicated groups to establish systems of greater equity and justice in the provision of vaccines to the world. (Privor-Dumm et al. 2023) One advocated alternative is a multilateral system in which the failures at various stages of vaccine development are analyzed, business principles are used to propose ways to raise the standard of vaccine research and development and the principle of “responsibility to protect” is used as an ethical mode of vaccine development. (Jecker et al. 2023).

One of the ways proposed to address inequities in the COVID-19 pandemic is to study the increase in access to and supply of additional green spaces, spaces not sufficiently available to the poor, the poorly educated and racial minorities. (Gao et al. 2023) The high prevalence of COVID in areas with high density supports the need to assess green space provisions. (Haase 2020), a concept referred to by other authors as primarily the need to encourage “social distancing,” something that high-risk patients often cannot obtain. (Silva and Smith 2020) The developing world is not devoid of studies as the abundant research activities are outlined in the articles by Shadmi and Parker illustrate. (Parker et al. 2021) (Shadmi et al. 2020).

In summary, the arena of research ethics includes new aspects of informed consent, deferred ethics, and the need to contend with technology increasingly promoted during the outbreak such as COVID-tracking apps. A national research agenda addressing issues related to crime and justice in the COVID-19 area is recommended by criminologists. (J. M. Miller and Blumstein 2020).

## Further societal ethical issues

A category of ethical issues that defies easy categorization and might be considered societal ethical issues are those identified by the author Fine in his book *On Medicine as Colonialism*. He decries that loss of autonomy among providers, and the attendant increasing power of administrative agencies and pharmaceutical companies. The influence of the latter groups amplified significantly during the COVID-19 outbreak. The advent of new vaccines and pharmaceuticals was often under the directive of impersonal national and international agencies and organization. The upshot is the further diminution of the role of individual providers in clinical medicine. The fact that pharmaceutical companies supplied one-third of all vaccines elucidated and exacerbated this trend in American society. (Fine 2023).

## Prevention of future outbreaks and preparedness

Prevention is the key to stopping further outbreaks, and the ethical issues associated with this include the limited public health funding and the tendency of some administrations to reduce or eliminate pandemic funding prior to the outbreak. Should society start to mandate increased preventive program funding, and how can the work on pandemic preparedness be boosted in society-at-large? The editors of Nature appropriately felt three years ago that the time to prepare for the next outbreak was then. (Editorial staff 2020).

The role of many other disciplines will help dictate the future of COVID, its prevention and prevention of future such outbreaks. One essayist emphasizes the need for a landscape ecology approach and the need for a holistic approach including specialists, such as climatologists, biologists, anthropologists, and environmental economic historians. (Ziegler 2016) The authors emphasize the importance of urban green infrastructures in combatting outbreaks. (Azevedo et al. 2020).

The need to understand the balance between medical and economic controls in containing an outbreak is essential. (Bavli et al. 2020) The adverse medical and societal aspects associated with an economic recession are considerable and hard to quantify. The COVID-19 pandemic, for example had a greater impact on the economy than the 2008/9 recession, Lockdown were associated with increased domestic violence and a greater delays in receiving medical care. The closure of schools is known to show a decline in educations achievements. Children living in poverty, those with special needs, and those who speak a different language at home are most impacted by a pandemic. (Hoofman and Secord 2021).

Pandemic preparedness must include the knowledge acquired by ethicists during this outbreak, and experiences in areas such as allocation of resources, will help in providing future care. (O’Sullivan et al. 2022) The spectrum of ethical dilemmas and challenges in disaster scenarios provide the ability to develop supportive guidelines for training programs during future events. (Dittborn et al. 2022) One is reminded of the quote of Marcus Aurelius, “Do not act as if you are going to live ten thousand years,” (Marcus Aurelius 160AD) Our needs for pandemic preparedness are immediate and reminiscent to the frequency with which 100-year climatic events are occurring with greater frequency.

Other authors have stressed the need for solidarity in our approach to pandemics. (Pascoe and Stripling 2020) The concept of mandatory vaccination, a highly disputed policy discussed above, is discussed by other public health authorities in the context of solidarity. (Yeh 2022).

The concept of COVID-19 reparations is also considered by some a means to achieve greater equity and will be needed before a future COVID-19 like outbreak can be prevented. (Rosenthal and Caplan 2021) A selective attention to women’s needs is also mentioned in the need for full equity. (Evans et al. 2021).

A group of British and Welsh investigators early in the outbreak reviewed commonly used ethical guidelines and developed a set of principles needed for the current and future outbreaks. These include the traditional concepts of respect, fairness, minimizing harm, reciprocity (supporting those who take on the work of the outbreak), and proportionality as discussed above. Other important concepts include flexibility, an ability to work together, inclusiveness, and communication. The public often do not understand scientific methodology including the fact that guidelines change through time. Other concepts outlined are transparency, reasonability, responsibility and accountability—both professionals and patients. This broad and perhaps overly extensive set of principles overlap among themselves but provide a corps for assessing programs and their efficacy. (H. Smith et al. 2021).

Governmental aspects needed for the current and future outbreaks include linkage of data as part of digital literacy, (The Lancet Digital Health 2022) and also the need to ensure that emergency orders comply with human rights obligations (Sun et al. 2022) An analysis of vaccination data, for example, showed that over 14 million lives were saved during the first year of vaccine availability, but the extent of this finding in the developing world and the complains with human rights in poor countries appears to be limited. (Watson et al. 2022).

Future approaches to the outbreak may entail the use of gene editing CRISPR technology to enhance immunity, recognizing the protected and preferred immunity of many individuals (a phenomenon to date not well understood). Such interventions will interface the many ethical issues attendant with somatic gene therapy. (Germani et al. 2021).

It was argued early in the COVID-19 outbreak that what we now face is a consequence of insufficient research (on pandemics, ecology of markets, diagnostics), in appropriate education systems (borne out by recent US educational system data), and globally fragile health structures and human services. (Escotet 2020) A major review of pandemics cited earlier concludes that only with adequate research in preventing pandemics will we be able to “avoid the extreme burden on healthcare systems.” (Sampath et al. 2021) A related editorial in the New York Times by a public health professional reminds us that the only way to prevent the next epidemic is to improve surveillance (compromised when only 2.9% of the US federal health care expenses goes to public health) (Kamal and Hudman 2020), enlarge the global health care work force, and ensure equitable distribution of vaccines and therapeutics. (Spencer 2022). All such factors confront the issues of equity and distributive justice.

Prevention and preparedness for the developing world is equally intense. Diagnostics and vaccinology are particular fields where support to the developing world is important and where the sense of global equity is shown to be most distressed. Diagnostic testing in the developing world in an analysis of 86 countries with available data showed that the Human Development Index, and the size of urban populations were the two variables most significantly correlated with testing. (Marziali et al. 2021) Africa especially from the start needed intergovernmental cooperation, a sense of solidarity with the advanced nations which produce the diagnostics, and continued domestic production of test kits (started in Kenya, Morocco, Senegal, and South Africa) to continue to control the outbreak. (Nkengasong 2020).

The host of ethical issues attendant with vaccines in the developing world include the dilemmas of using Africa nations for COVID trials (as occurred with HIV early in that outbreak), (Lurie 2021) The sense often exists that the advanced economies of the world are spending funds on continued boosters when developing world are struggling to get their first dose of vaccines, (Maxmen 2021) Other issues include the possible benefits yet attendant controversies associated with cash transfers for vaccines, (Arezki 2021) the need for increased approval of COVID-19 vaccines in the developing world, (Kozlov 2021) and the needs for comparator trials in the developing world as new variants emerge. Many of the SARS-2 variants may first be identified in low and middle income countries (Brazil for example had over 20 variants associated with the gamma variants emerging in 2021). (Levi et al. 2021) African nations are notably absent from COVID trials, another example of disturbed equity in COVID ethics. (Roussi and Maxmen 2020) Antiviral supplies (Tsanni 2021) and production face many of the same hurdles as vaccines (Bajaj and Stanford 2022).

The production of vaccines needs to be carried out in a type of “patent diplomacy.” This is promoted in India and South Africa, as a means of waiving Trade-Related Intellectual Property Rights (TRIPS) in order to share technology on vaccines and pharmaceuticals. Such concepts will help supply the world’s poor with their needs and fulfill the perceived moral obligations of the world’s rich to the world’s poor. (Singh et al. 2023) A hopeful element is the Lancet Commission for COVID-19 task force which is establishing recommendations to achieve access, justice and equity for vaccines and therapeutics. The commission is also engaged in combating anti-science and in establishing collaborative research networks. (Hotez et al. 2021).

A number of international organizations work toward establishing equity in the developing world, including COVID-19 Vaccines Global Access (COFVAX) and work of the Global Alliance for Vaccines and Immunizations. These organization emphasize the need to establish equity and the importance of including refugees and asylum seekers in services. (Manirambona et al. 2021).

## Summary

COVID ethics can be seen from a variety of perspectives. Although COVID epidemiology is relatively unique with respect to its infectivity, its relationship to social media and political party persuasion, and the inhumane impact on patients and their families in their dying moments, it is not completely unique in respect to the conventional clinical and societal ethics associated with it. The main issue society faces at the present is trying to prevent future pandemics with similar or worse morbidity and mortality. Such prevention will entail particular attention to the ecologic sciences, comparative epidemiology and social sciences which assess the impacts of social media and politics on the management of outbreaks.

## Methods used for this manuscript

As an editor for a major textbook’s online edition (Papadakis et al. 2020) the author reviewed medical literature weekly to monthly for the duration of the height of the outbreak from spring, 2020, to the present (truncated at the summer, 2022, for this talk and article). The methods included attention at the start of the outbreak with PubMed to the only journal providing widespread coverage of the COVD-19 outbreak including British and American clinical journals (Lancet, BMJ, NEJM, AIM, CID), scientific journals (Science, PNAS, JID, Cell series), public health reports (MMWR, WHO reports), and on-line data bases (Johns Hopkins tally of data). Google Scholar was used in addition to identify the most prominent and highly cited articles regarding ethics associated with COVID-19. Searches from PubMed were limited using the “Best Match” command, a mode of limiting results based on past usage, publication date, relevance score and type of article, (Fiorini et al. 2018). From the hundreds of reviewed articles during the first three years of the outbreak, 67 articles were identified as showing particular attention to ethical issues.

A second method included PubMed searches for a systematic review using as search commands (COVID-19 AND (equity OR justice OR autonomy OR beneficence OR nonmaleficence OR vaccine hesitance(y) OR duty to care OR transparency OR solidarity OR [developing world OR lower and middle income countries (LMIC)]). The ranking system available for PubMed was used to pick the top 25 articles when the number exceeded 25 for a given search. Only articles in English or with English translation were used. The time command was the interval March, 2020 through January 2024. The number of unique articles (not included in the above method) added by this second method was 128.^7^

## Data Availability

Readers can contact me directly at w.shandera@gmail.com for data set information

## Footnotes

1 The complex relationships of ethical issues are typified by the remarks of the Indian academician Gopichandran who considers the “forest of recommendations (masks, hygiene, distancing, quarantine, isolation) with the “trees” of individual patients and their ethical issues (Gopichandran 2020).

2 The importance of knowing clinical epidemiology and the past history of quarantines, pandemics and history of hospitals in relation to epidemics was also emphasized in a review published during the early years of the COVID-19 outbreak (Bassareo et al. 2020).

3 These include a text from Japan, The Ethics of Pandemics (Hirose 2023), a book that appeared early in the outbreak (Schwartz 2020), a work that encompasses the field of critical feminist phenomenology (Butler 2022), and books on pandemic ethics by Bramble and ten Haave. (Bramble 2020) (ten Haave 2022).

4 Bramble posed a set of questions early in the outbreak which included how long we could ethically maintain the lockdown, but thought the real victims were the elderly who were most susceptible to the disease and the economically disadvantaged who were forced to prematurely return to work.

5 This is akin to problems with computer literacy in patients with certain long-standing illnesses such as chronic kidney disease (CKD), a vulnerable group whose care may in particular be aided with telehealth. (Natale et al. 2023).

6 Traditional measures used to assess public health activities are often quantitative and emphasize life regardless of quality. Two popular touted measures are Quality Adjusted Life Years (QALYs) and Disability Adjusted Life Years (DALYs). But their application in the arena of COVID-19 infections shows the limitation of such measures. (Reddy 2020).

7 A publication looking at search terms related to health governance in which similar but broader concepts were used (eg, “structural changes, approach to inequity”) showed a high degree of interconnection in health equity topics. (Kheir-Mataria et al. 2022).

## Declarations

### Informed consents

There were no informed consents involved, and being an independent one-author review.
